# KIFC1 is Associated With Sarcomatoid Differentiation, Immune Response, and a Poor Prognosis in Clear Cell Renal Cell Carcinoma

**DOI:** 10.1002/cam4.71687

**Published:** 2026-02-26

**Authors:** Yoshinori Nakano, Yohei Sekino, Go Kobayashi, Hikaru Nakahara, Shintaro Akabane, Kenshiro Takemoto, Miki Naito, Shunsuke Miyamoto, Kohei Kobatake, Hiroyuki Kitano, Keisuke Goto, Akihiro Goriki, Keisuke Hieda, Takao Hinoi, Nobuyuki Hinata

**Affiliations:** ^1^ Department of Urology, Graduate School of Biomedical and Health Sciences Hiroshima University Hiroshima Japan; ^2^ Laboratory of Molecular Pathology, Department of Molecular Biosciences Radiation Effects Research Foundation Hiroshima Japan; ^3^ Department of Clinical and Molecular Genetics Hiroshima University Hospital Hiroshima Japan; ^4^ Department of Gastroenterological and Transplant Surgery, Graduate School of Biomedical and Health Sciences Hiroshima University Hiroshima Japan

**Keywords:** centrosome clustering, epithelial–mesenchymal transition, immune response, KIFC1, renal cell carcinoma

## Abstract

**Introduction:**

Centrosome clustering is a cancer‐specific adaptation that allows cells with centrosome amplification to evade mitotic catastrophe and has emerged as a potential therapeutic target. We analyzed the prognostic role of several molecules related to centrosome clustering and found that Kinesin Family Member C1 (*KIFC1*) was strongly associated with a poor prognosis. KIFC1, a kinesin motor protein, plays a central role in centrosome clustering. However, its biological and clinical significance in clear cell renal cell carcinoma (ccRCC) remains poorly understood.

**Methods:**

We conducted a comprehensive analysis using several public datasets (TCGA KIRC, JAVELIN101, IMmotion151, and others) and a Hiroshima ccRCC cohort (*n* = 110) to evaluate the expression of *KIFC1*, clinicopathological associations, the prognosis, and treatment response. Gene Set Enrichment Analysis was performed to explore associated pathways.

**Results:**

Immunohistochemical and *in silico* analyses showed that high *KIFC1* expression was significantly associated with high tumor grade, advanced TNM stage, and sarcomatoid differentiation. A multivariate analysis demonstrated that the high‐expression of *KIFC1* was independently associated with poor overall survival. Gene Set Enrichment Analysis revealed enrichment of epithelial–mesenchymal transition and interferon gamma response pathways in *KIFC1*‐high tumors. The expression of *KIFC1* was also correlated with TKI resistance, immune response, high clonal neoantigen load, and *BAP1* mutation.

**Conclusion:**

*KIFC1* serves as a multifunctional molecule linking epithelial–mesenchymal transition, immune modulation, and treatment resistance. It may be a promising prognostic biomarker and therapeutic target in ccRCC, warranting further functional and clinical investigation.

## 1 Introduction

Renal cell carcinoma (RCC) represents the most fatal form of kidney malignancy, accounts for 2% of global cancer diagnoses and deaths [1]. Among its various subtypes, clear cell RCC (ccRCC) is the most prevalent, accounting for approximately 70%–75% of cases [2]. Furthermore, between 20% and 35% of RCC patients present with metastatic disease at the diagnosis, while 20%–40% of patients who undergo early nephrectomy eventually experience recurrence with metastatic progression [3, 4, 5].

In recent years, pharmacological interventions for unresectable or metastatic RCC have undergone significant advancements, particularly with the advent of molecularly targeted therapies, such as tyrosine kinase inhibitors (TKIs) and immunotherapies utilizing immune checkpoint inhibitors (ICIs). Although these therapeutic strategies have markedly improved clinical outcomes [6, 7], the cure rate is still only approximately 10% [7, 8], which underscores the inadequacy of current treatment efficacy. Additionally, these therapies are associated with a substantial physical burden and high medical costs. Therefore, there is an urgent need for the development of novel therapeutic agents that leverage alternative mechanisms of action against metastatic RCC [9].

Centrosomes are small intracellular organelles composed of a pair of centrioles surrounded by a structured layer of pericentrosomal material [10, 11]. Regulation of the number of centrosomes is tightly controlled throughout the cell cycle, with the two centrosomes present during mitosis serving as pivotal anchors for each pole of the bipolar mitotic spindle, ensuring the accurate segregation of chromosomes into newly formed daughter cells [12].

Aberrations in centrosome structure and number, particularly centrosome amplification, are frequently observed in cancer, including RCC [13, 14]. When cells enter mitosis with centrosome amplification, they typically form a multipolar mitotic spindle, a condition that—if uncorrected—invariably results in cell death. However, to evade lethality, cancer cells employ several compensatory mechanisms, the most well‐characterized of which is centrosome clustering [15, 16]. This cancer‐specific adaptation has been proposed as a promising target for novel therapeutic strategies and has also been implicated in resistance to various anticancer agents [17]. Centrosome clustering was first observed in mouse neuroblastoma cells approximately 30 years ago [18]. This process allows cancer cells to maintain viable bipolar divisions.

Recent studies suggest that centrosome clustering may be a cyst‐selective therapeutic target to improve the renal morphology and function in autosomal dominant polycystic kidney disease [19]. On the other hand, centrosome abnormalities in RCC, especially in relation to centrosome amplification and centrosome clustering, remain poorly understood. Several molecules have been reported to be involved in the formation of centrosome clustering [20], and there is room for further investigation as to which of these molecules may be promising therapeutic targets in RCC.

In this study, we comprehensively searched for molecules related to centrosome clustering and identified Kinesin Family Member C1 (*KIFC1*) as a poor prognostic factor through a comprehensive analysis using multiple public databases. The expression of *KIFC1* was confirmed by immunohistochemistry, and a prognostic analysis was also performed in our own cohort. Furthermore, *in silico* analysis was performed to examine the association with clinical pathological factors and related signaling pathways, and the functional role of *KIFC1* in ccRCC and its potential as a therapeutic target were evaluated.

## 2 Methods

### 2.1 Tissue Samples

A total of 110 tumors were collected from patients pathologically diagnosed with ccRCC who underwent nephrectomy at Hiroshima University Hospital (Hiroshima, Japan). The medical records of patients at Hiroshima University Hospital between April 1999 and May 2019 were retrospectively reviewed. The TNM classification system was used to determine the tumor stage. The Institutional Review Board of Hiroshima University approved this study (Hiroshima University).

### 2.2 Immunohistochemistry

IHC for KIFC1 (anti‐KIFC1 antibody, 1:100, H00003833‐M01, Abnova, Taipei, Taiwan) was performed on representative tumor blocks using an automated immunostainer (Bond‐3, Leica Biosystems) according to the manufacturer's protocol. Samples were considered positive when > 10% of tumor cells were stained. Further details of the IHC procedure are provided in the Methods in Appendix S1.

### 2.3 Public Databases

UCSC Xena was used to determine the *KIFC1* expression in The Cancer Genome Atlas (TCGA) KIRC dataset (https://xena.ucsc.edu). The processed expression array data were downloaded from Gene Expression Omnibus and Array Express. The processed data from several studies were downloaded. The accession numbers and studies are summarized in Table S1.

The RNA sequence data and clinical data of IMmotion 150 (EGAC00001000946) and IMmotion 151 (EGAC00001001813) were downloaded from the European Genome–phenome Archive (https://ega‐archive.org/).

### 2.4 Statistical Analysis

Statistical analyses were performed using appropriate tests, including Student's *t*‐test, the Mann–Whitney *U* test, one‐way ANOVA, and a log‐rank test for survival analysis. *p* values of < 0.05 were considered statistically significant. Further details are provided in the Methods in Appendix S1.

### 2.5 Gene Set Enrichment Analysis

Gene Set Enrichment Analysis (GSEA) was performed using the GSEA software downloaded from the Broad Institute website (https://www.gsea‐msigdb.org). Samples were stratified into *KIFC1*‐high and *KIFC1*‐low groups based on the median expression level of *KIFC1*. The gene set collection “h.all.v2024.1.Hs.symbols.gmt” from the Molecular Signatures Database (MSigDB) was used for the analysis. The following phenotype labels were assigned: “*KIFC1*‐high” and “*KIFC1*‐low.” The number of permutations was set to 1000, and permutation type was set to “phenotype.” Enrichment score, normalized enrichment score (NES), nominal *p* value, and false discovery rate *q*‐value (FDR *q*‐value) were calculated. Gene sets with FDR *q*‐values of < 0.05 were considered statistically significant. Gene sets that met the less stringent criterion of FDR *q*‐value < 0.25, while not statistically significant, were identified as exploratory candidates for generating biologically relevant hypotheses.

## 3 Results

### 3.1 Association Between Centrosome Clustering‐Related Molecules and the Prognosis in Clear Cell Renal Cell Carcinoma

**FIGURE 1:**
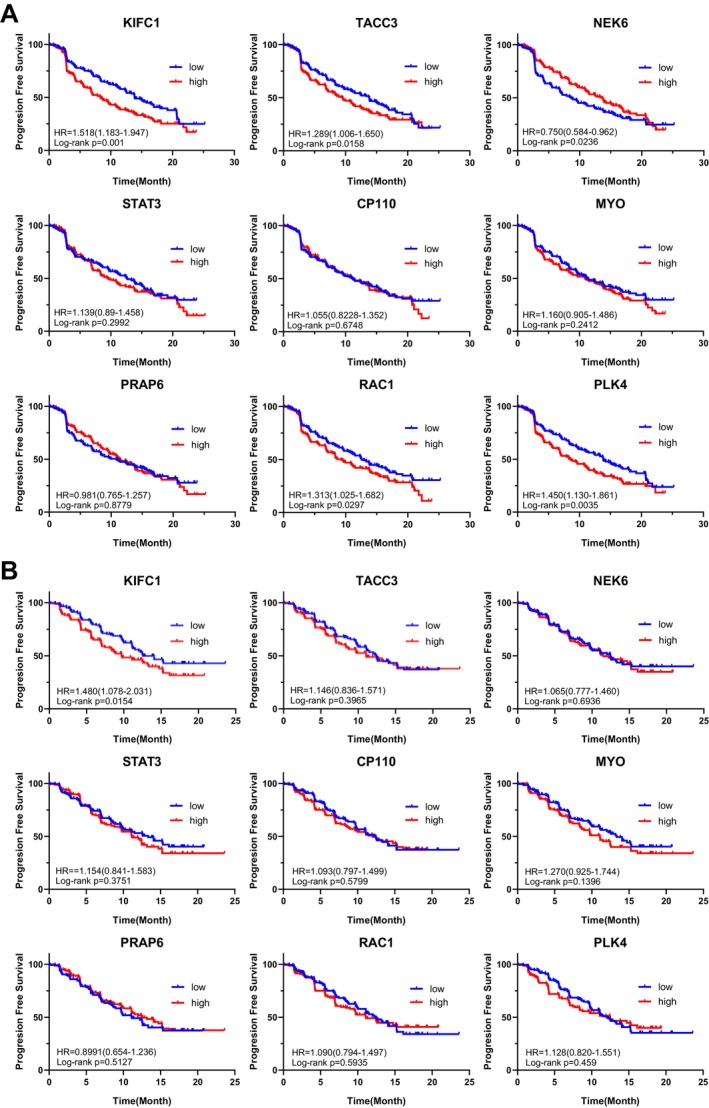
Prognostic impact of centrosome clustering‐related molecule expression in clear cell renal cell carcinoma patients treated with TKI monotherapy or immune‐oncology‐TKI combination therapy. (A) Kaplan–Meier curves for progression‐free survival (PFS) in RCC patients from the IMmotion151 trial (TKI monotherapy arm), stratified by high (red, *n* = 203) versus low (blue, *n* = 204) expression of centrosome clustering‐related genes (KIFC1, TACC3, NEK6, STAT3, CP110, MYO, PRAP6, RAC1, and PLK4). Hazard ratios (HR), 95% confidence intervals (CI), and log‐rank *p*‐values are indicated for each gene. (B) Kaplan–Meier curves for PFS in RCC patients from the JAVELIN101 trial (IO‐TKI combination arm), stratified by high (red, *n* = 173) versus low (blue, *n* = 179) expression of the same genes as in (A). HRs, 95% CIs, and log‐rank *p*‐values are shown for each gene. Among the nine analyzable centrosome clustering‐related molecules, high *KIFC1* expression showed the strongest association with poor prognosis in both treatment arms.

To investigate the relationship between the expression levels of centrosome clustering‐related molecules and the prognosis in ccRCC, we analyzed datasets from phase III clinical trials comparing TKI monotherapy with immune‐oncology‐TKI combination therapy (IMmotion151, JAVELIN101). Among the centrosome clustering‐related molecules reported by Sabat‐Pospiech et al. [20], we analyzed nine candidates (*KIFC1, TACC3, NEK6, STAT3, CP110, MYO, PRAP6, RAC1*, and *PLK4*). Among these, *KIFC1* exhibited the highest hazard ratio in ccRCC and showed the strongest association with a poor prognosis. Specifically, the hazard ratio was 1.518 in the IMmotion151 trial and 1.480 in the JAVELIN101 trial, and in both cohorts, high *KIFC1* expression was significantly associated with a poor prognosis (Figure 1A,B). The kinesin‐like protein KIFC1, a nonessential minus end‐directed motor of the kinesin‐14 family, plays a pivotal role in maintaining microtubule system stability [21]. As a minus‐end‐directed motor protein, KIFC1 facilitates centrosome clustering during mitosis and serves as a key regulator of this process [20, 21, 22, 23]. The molecular and cytological significance of KIFC1 has been extensively documented, with high‐expression levels linked to a poor prognosis in multiple solid tumors [24, 25, 26, 27, 28]. Based on these findings and the results of the analyses, this study focused on KIFC1 and integrated key clinicopathological factors, including the prognosis and immune cell infiltration. This study aimed to elucidate the functional significance of the *KIFC1* expression in ccRCC through comprehensive analyses using multiple public databases.

### 3.2 Association Between *KIFC1* Expression, Clinicopathological Features, and the Prognosis in Clear Cell Renal Cell Carcinoma

**FIGURE 2:**
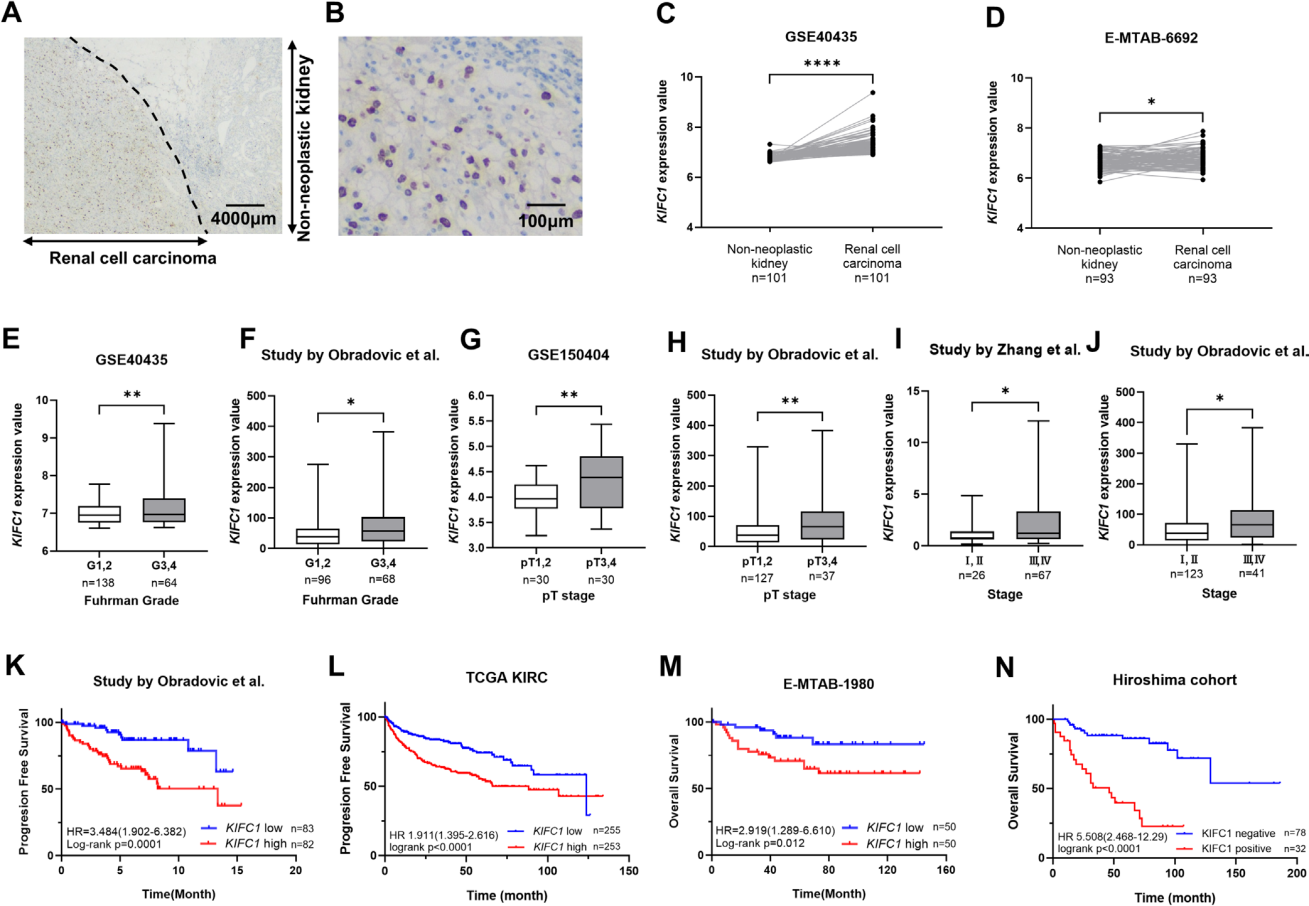
Association between KIFC1 expression, clinicopathological features, and prognosis in ccRCC. (A) Representative immunohistochemical staining of KIFC1 in RCC tissue and adjacent nonneoplastic kidney, showing stronger cytoplasmic staining in tumor cells compared to nonneoplastic tissue. (B) Higher magnification image of KIFC1 immunostaining in RCC tissue. (C, D) Comparison of KIFC1 mRNA expression between nonneoplastic kidney and RCC tissues in the GSE40435 (C) and E‐MTAB‐6692 (D) datasets. *KIFC1* expression is significantly elevated in RCC tissues. (E, F) Association between *KIFC1* expression and Fuhrman nuclear grade in GSE40435 (E) and Obradovic et al. (F) datasets. Higher *KIFC1* expression correlates with higher grade tumors. (G–J) Association between *KIFC1* expression and tumor stage in multiple datasets: GSE40435 (G), GSE150404 (H), Zhang et al. (I), and Obradovic et al. (J). Higher *KIFC1* expression is observed in more advanced stages. (K, L) Kaplan–Meier analysis of progression‐free survival according to *KIFC1* expression in the Obradovic et al. cohort (K), TCGA KIRC (L). High *KIFC1* expression is associated with shorter progression‐free survival. (M, N) Kaplan–Meier analysis of overall survival in the E‐MTAB‐1980 dataset (M), the Hiroshima cohort (N), showing significantly worse survival in KIFC1‐positive cases compared to KIFC1‐negative cases. Statistical significance is indicated as follows: *p < 0.05, **p < 0.01, ****p < 0.0001.

To assess the clinical significance of the expression of *KIFC1*, we subjected 110 ccRCC tissue samples to immunohistochemical analyses (Hiroshima cohort. Table 1). Weak to moderate KIFC1 staining was observed in nonneoplastic kidney tissue, whereas ccRCC tissues exhibited intense cytoplasmic staining (Figure 2A,B). Tumor samples were classified as KIFC1‐positive when > 10% of tumor cells showed staining. Overall, 32 of the 110 cases (29.1%) were KIFC1‐positive. Notably, KIFC1 positivity was significantly associated with higher nuclear grade and advanced T, N, and M stages (Table 1). We also analyzed public datasets from the TCGA and Gene Expression Omnibus. In the GSE40435 and E‐MTAB‐6692 datasets, the *KIFC1* expression in ccRCC tissues was significantly higher than that in nonneoplastic kidney tissues (Figure 2C,D). The analysis of the *KIFC1* expression in relation to Fuhrman grade using GSE40435 and the study by Obradovic et al. showed a positive association between high *KIFC1* expression and higher grade (Figure 2E,F). Furthermore, high *KIFC1* expression was correlated with advanced tumor stage in multiple datasets, including GSE40435, GSE150404, as well as the studies by Zhang et al. and Obradovic et al. (Figure 2G–J). To evaluate the prognostic impact of *KIFC1* expression, we performed a Kaplan–Meier survival analysis. In the cohort by Obradovic et al. and TCGA KIRC cohort, ccRCC patients with high *KIFC1* expression levels had significantly shorter progression‐free survival than those with low‐expression levels (Figure 2K,L). Similarly, in both the E‐MTAB‐1980 dataset and the Hiroshima cohort, overall survival was significantly worse in KIFC1‐positive cases than in KIFC1‐negative cases (Figure 2M,N). Importantly, these four cohorts received various treatment regimens, indicating that high *KIFC1* expression is associated with a poor prognosis regardless of therapeutic approach. Finally, univariate and multivariate Cox proportional hazards analyses were performed to assess the prognostic value of *KIFC1*. In the multivariate analysis, high *KIFC1* expression was independently associated with poorer overall survival (hazard ratio: 3.194; 95% confidence interval: 1.419–7.188; *p* = 0.005), along with pT stage and M stage (Table 2). Furthermore, a multivariate analysis using TCGA KIRC data showed that high *KIFC1* expression was an independent prognostic factor for progression‐free survival (Table 3).

**TABLE 1:** Hiroshima cohort patient characteristics.

		KIFC1 expression				
		Positive		Negative		*p*
		(*n* = 32)	(%)	(*n* = 78)	(%)	
Age						
< 65	(*n* = 53)	11	34.4	42	53.8	0.062
> 65	(*n* = 57)	21	65.6	36	46.2	
Sex						
Female	(*n* = 28)	5	15.6	23	29.5	0.117
Male	(*n* = 82)	27	84.4	55	70.5	
T stage						
T1, 2	(*n* = 78)	16	50	62	79.5	0.0025
T3, 4	(*n* = 32)	16	50	16	20.5	
N stage						
N0	(*n* = 102)	25	78.1	77	98.7	0.0003
N1, 2	(*n* = 8)	7	21.9	1	1.3	
M stage						
M0	(*n* = 92)	20	83.6	72	92.3	0.0003
M1	(*n* = 18)	12	16.4	6	7.7	
Fuhman grade						
G1, 2	(*n* = 78)	14	43.8	64	82.1	< 0.001
G3, 4	(*n* = 32)	18	56.2	14	17.9	
Sarcomatoid change						
Negative	(*n* = 107)	30	93.7	77	98.7	0.172
Positive	(*n* = 3)	2	6.3	1	1.3	

*Note:* Clinicopathological features of patients stratified by KIFC1 expression status in the Hiroshima cohort. Categorical variables were compared using Fisher's exact test as appropriate. Patients with positive KIFC1 expression showed significantly higher rates of advanced T stage (T3–4), nodal metastasis (N1–2), distant metastasis (M1), and higher Fuhrman grade (G3–4). Sarcomatoid change and other variables showed no significant difference.

**TABLE 2:** Univariate and multivariate Cox regression analyses in the Hiroshima cohort.

	Univariate analysis		Multivariate analysis	
	HR (95% CI)	*p*	HR (95% CI)	*p*
T Stage				
T1, 2	1 (Ref.)	< 0.001	1 (Ref.)	0.0099
T3, 4	5.364 (2.674–10.763)		2.799 (1.281–6.116)	
M stage				
M0	1 (Ref.)	< 0.001	1 (Ref.)	0.0069
M1	10.755 (4.969–23.278)		3.247 (1.383–7.624)	
Fuhman grade				
G1,2	1 (Ref.)	0.0001	1 (Ref.)	0.075
G3,4	3.917 (1.938–7.914)		1.986 (0.933–4.229)	
KIFC1				
Negative	1 (Ref.)	< 0.001	1 (Ref.)	0.005
Positive	5.508 (2.468–12.29)		3.194 (1.419–7.188)	

*Note:* Univariate and multivariate Cox proportional hazards analyses of factors associated with overall survival. In multivariate analysis, advanced T stage (T3–4) and positive KIFC1 expression remained significant independent prognostic factors (hazard ratio: 3.194; 95% confidence interval: 1.419–7.188; *p* = 0.005). Hazard ratios (HRs), 95% confidence intervals (CIs), and *p*‐values are shown.

Abbreviations: CI, confidence interval; HR, hazard ratio.

**TABLE 3:** TCGA KIRC univariate and multivariate Cox regression analyses.

	Univariate analysis		Multivariate analysis	
	HR (95% CI)	*p*	HR (95% CI)	*p*
Age				
< 65	1 (Ref.)	0.062	1 (Ref.)	0.127
> 65	1.351 (0.986–1.852)		1.448 (0.900–2.329)	
Sex				
Female	1 (Ref.)	0.0137	1 (Ref.)	0.247
Male	1.551 (1.094–2.199)		1.323 (0.824–2.123)	
T stage				
T1, 2	1 (Ref.)	< 0.001	1 (Ref.)	0.0015
T3, 4	4.386 (3.154–6.099)		2.211 (1.354–3.613)	
N stage				
N0	1 (Ref.)	0.0002	1 (Ref.)	0.021
N1, 2	3.486 (1.789–6.790)		2.857 (1.171–6.969)	
M stage				
M0	1 (Ref.)	< 0.001	1 (Ref.)	< 0.001
M1	8.725 (6.278–12.130)		8.588 (5.128–14.382)	
Fuhman grade				
G1, 2	1 (Ref.)	< 0.001	1 (Ref.)	0.342
G3, 4	3.329 (2.283–4.853)		1.307 (0.752–2.271)	
Neoadjuvant therapy				
No	1 (Ref.)	0.0003	1 (Ref.)	0.0066
Yes	0.246 (0.115–0.526)		0.147 (0.036–0.586)	
KIFC1 expression				
Low	1 (Ref.)	< 0.001	1 (Ref.)	0.0057
High	1.938 (1.397–2.689)		2.127 (1.246–3.629)	

*Note:* Univariate and multivariate Cox proportional hazards regression analyses for progression‐free survival in the TCGA KIRC cohort. Variables including age, sex, T stage, N stage, M stage, Fuhrman grade, neoadjuvant therapy, and *KIFC1* expression were evaluated. In multivariate analysis, advanced T stage (T3–4), lymph node metastasis (N1–2), distant metastasis (M1), absence of neoadjuvant therapy, and high *KIFC1* expression were independently associated with poorer disease‐free survival. Hazard ratios (HRs), 95% confidence intervals (CIs), and *p*‐values are shown.

Abbreviations: CI, confidence interval; HR, hazard ratio.

### 3.3 High *KIFC1* Expression and Gene Set Enrichment Analysis

**FIGURE 3:**
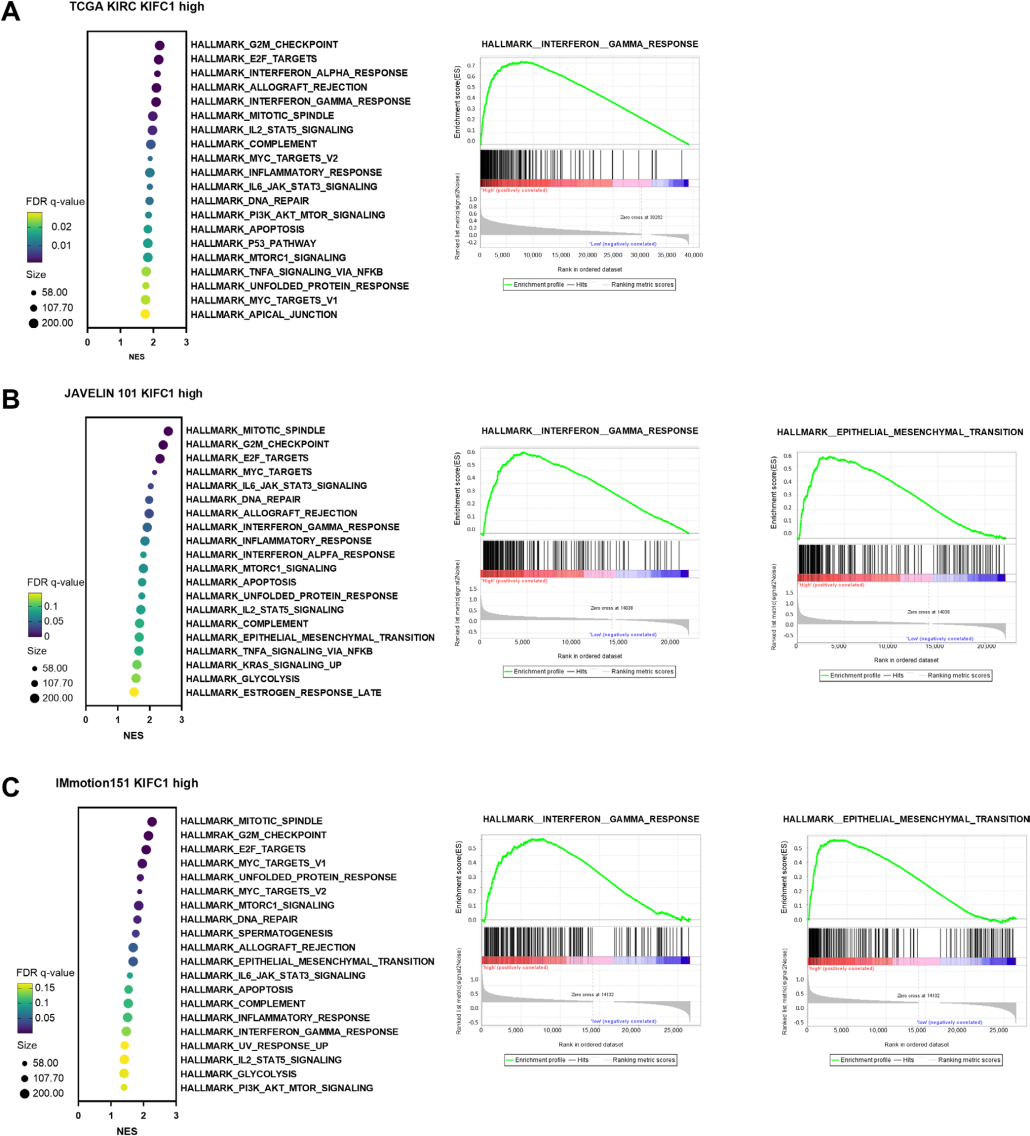
Gene set enrichment analysis of hallmark pathways associated with high KIFC1 expression in ccRCC across three independent datasets. (A) TCGA KIRC cohort (*n* = 187): Bubble plot (left) shows normalized enrichment scores (NES) and FDR *q*‐value for hallmark gene sets significantly enriched in tumors with high *KIFC1* expression. The interferon gamma response, G2M checkpoint, E2F targets, and allograft rejection pathways are among the most enriched. Representative enrichment plot (right) for the interferon gamma response gene set (NES > 2; FDR *q*‐value, 0.001). (B) JAVELIN101 cohort (*n* = 177): Bubble plot (left) demonstrates significant enrichment of proliferative and immune‐related pathways, including mitotic spindle, G2M checkpoint, interferon gamma response, and epithelial–mesenchymal transition (EMT) in tumors with high *KIFC1* expression. Representative enrichment plots (right) for interferon gamma response (NES, 1.92; FDR *q*‐value, 0.047) and EMT (NES, 1.67; FDR *q*‐value, 0.093) gene sets. (C) IMmotion151 cohort: Bubble plot (left) highlights enrichment of mitotic spindle, G2M checkpoint, E2F targets, interferon gamma response, and EMT pathways in tumors with high *KIFC1* expression. Representative enrichment plots (right) for interferon gamma response (NES, 1.47; FDR *q*‐value, 0.139) and EMT (NES, 1.68; FDR *q*‐value, 0.043) gene sets. In all cohorts, high *KIFC1* expression is associated with activation of immune/inflammatory pathways (notably interferon gamma response) and EMT, suggesting a link between *KIFC1* overexpression, immune modulation, and aggressive tumor phenotype. NES, normalized enrichment score.

To explore the biological pathways associated with high *KIFC1* expression in ccRCC, GSEA was performed using three independent datasets: TCGA KIRC, JAVELIN101, and IMmotion151. Hallmark gene sets were analyzed across all three datasets to identify pathways significantly enriched in tumors with high *KIFC1* expression. In the TCGA KIRC dataset, high *KIFC1* expression was positively correlated with several immune‐related and proliferative pathways, including G2M checkpoint, E2F targets, allograft rejection, and interferon gamma response (Figure 3A). Notably, the interferon gamma response gene set exhibited strong enrichment (NES, > 2; FDR *q*‐value, 0.001), suggesting an activated inflammatory or immune microenvironment in *KIFC1*‐high tumors. In the JAVELIN101 dataset, we analyzed 177 samples from the high *KIFC1* expression group in the immune‐oncology‐TKI combination therapy group. Similarly, enrichment was observed in proliferative pathways such as mitotic spindle and G2M checkpoint, as well as in interferon gamma response and epithelial–mesenchymal transition (EMT). Enrichment plots revealed a significant upregulation of interferon gamma response (NES, 1.92; FDR *q*‐value, 0.047) and an exploratorily significant upregulation of EMT (NES, 1.67; FDR *q*‐value, 0.093) hallmark gene sets in tumors with high *KIFC1* expression (Figure 3B). Consistent with these findings, the IMmotion151 dataset also demonstrated enrichment of mitotic spindle, G2M checkpoint, E2F targets, EMT (NES, 1.68; FDR *q*‐value, 0.043), and interferon gamma response (NES, 1.47; FDR *q*‐value, 0.139) in the *KIFC1*‐high group. These pathways are commonly associated with aggressive tumor behavior and immune modulation (Figure 3C). These consistent findings across multiple independent cohorts highlight that high *KIFC1* expression is closely associated with proliferation and the immune response in ccRCC.

### 3.4 Relationship Between *KIFC1* Expression and Sarcomatoid Differentiation

**FIGURE 4:**
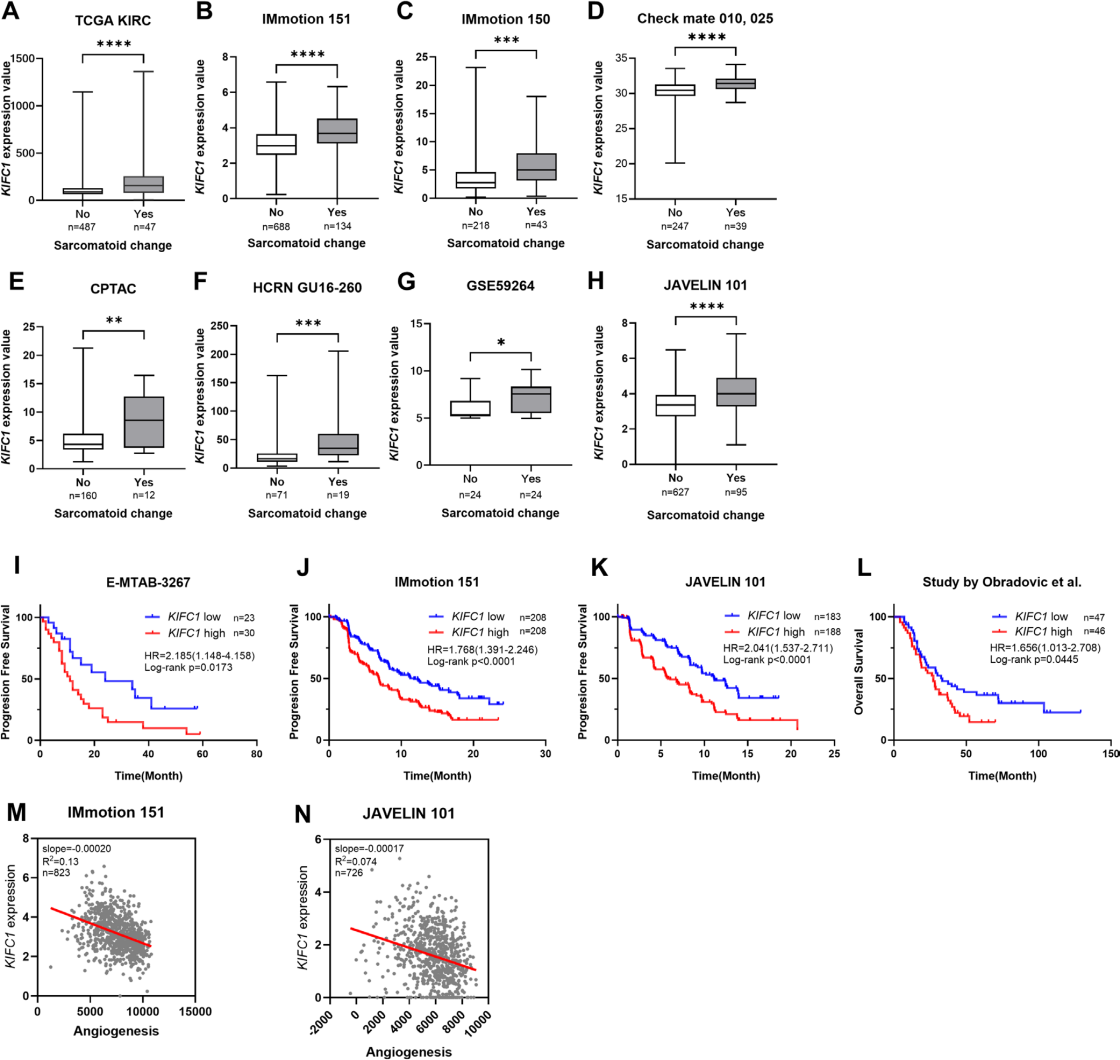
Association between KIFC1 expression, sarcomatoid differentiation, and prognosis in ccRCC. (A–G) Box plots showing *KIFC1* expression levels in RCC tumors with (Yes) or without (No) sarcomatoid change across multiple independent cohorts: TCGA KIRC (A), IMmotion151 (B), IMmotion150 (C), CheckMate 010/025 (D), CPTAC (E), HCRN GU16‐260 (F), and GSE59264 (G), JAVELIN101 (H). In all cohorts, KIFC1 expression is significantly higher in tumors exhibiting sarcomatoid differentiation. Statistical significance: *p < 0.05, **p < 0.01, ***p < 0.001, ****p < 0.0001. (I–L) Kaplan–Meier survival curves for progression‐free survival (PFS) or overall survival (OS) in RCC patients stratified by *KIFC1* expression (high vs. low) in four independent TKI‐treated cohorts: E‐MTAB‐3267 (I), IMmotion151 (J), JAVELIN101 (K), and Obradovic et al. (L) High *KIFC1* expression is consistently associated with significantly shorter PFS or OS, indicating poor prognosis. Hazard ratios (HR) and log‐rank *p*‐values are indicated in each panel. (M, N) Scatter plot showing a negative correlation between *KIFC1* expression and Angiogenesis in the IMmotion 151 cohort (*R* ^2^ = 0.13, *p* < 0.0001, *n* = 823) and JAVELIN 101 cohort (*R* ^2^ = 0.074, *p* < 0.0001, *n* = 726), indicating that high *KIFC1* expression is associated with TKI resistance.

The activation of the EMT pathway in the GSEA results described in Figure 3 suggested a potential link between high *KIFC1* expression and sarcomatoid differentiation. To further explore this relationship, we analyzed the association between the expression of *KIFC1* and sarcomatoid changes in eight independent cohorts: TCGA KIRC, IMmotion151, IMmotion150, CheckMate 010/025, CPTAC, HCRN GU16‐260, GSE59264, and JAVELIN101. We found a significant correlation between high *KIFC1* expression and the presence of sarcomatoid differentiation across all cohorts (Figure 4A–H). Sarcomatoid features in RCC have been previously reported to be correlated with a poor response to TKIs and an unfavorable prognosis [29, 30, 31]. Therefore, we evaluated the prognostic impact of the *KIFC1* expression in ccRCC patients receiving standardized treatment regimens. In four independent cohorts (E‐MTAB‐3267, IMmotion151, JAVELIN101, and Study by Zhang et al.) treated with TKI monotherapy, high *KIFC1* expression was consistently correlated with shorter progression‐free survival and overall survival (Figure 4I–L). A recent study has shown that increased *KIFC1* expression may contribute to the activation of EMT‐related transcriptional programs through *HMGA1* [32]. In addition, *KIFC1* has been reported to interact with *MYH9*, thereby physically and signaling‐wise supporting cytoskeletal remodeling and mesenchymal phenotypic changes during EMT [33]. Consistently, in the TCGA KIRC cohort, *KIFC1* expression showed significant positive correlations with *HMGA1* (*R* ^2^ = 0.089, *p* < 0.001) and *MYH9* (*R* ^2^ = 0.058, *p* < 0.001) (Figure S1A,B). These findings support the notion that *KIFC1* upregulation is associated with EMT activation and may contribute to sarcomatoid differentiation in ccRCC. Furthermore, we evaluated the correlation between the expression of *KIFC1* and angiogenesis signatures using the IMmotion151 and JAVELIN101 cohorts. A significant negative correlation was observed in both cohorts (IMmotion151: *R* ^2^ = 0.013, *p* < 0.001; JAVELIN101: *R* ^2^ = 0.074, *p* < 0.001) (Figure 4M,N). These findings suggest that the overexpression of *KIFC1* may be associated with resistance to TKIs and could potentially serve as a biomarker for guiding therapeutic decisions in ccRCC.

### 3.5 Relationship Between *KIFC1* Expression and the Immune Response

**FIGURE 5:**
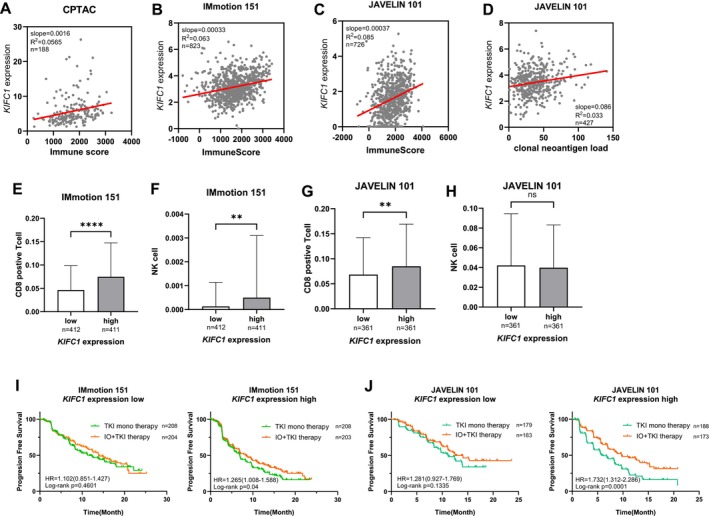
Relationship between KIFC1 expression, immune activation, and therapeutic response in renal cell carcinoma (RCC). (A–C) Scatter plot showing a positive correlation between KIFC1 expression and Immune Score in the CPTAC cohort (*R* ^2^ = 0.0565, *p*‐value = 0.001, *n* = 188), IMmotion 151 (*R* ^2^ = 0.013, *p* < 0.001, *n* = 823), and JAVELIN 101 (*R* ^2^ = 0.074, *p* < 0.001, *n* = 726), indicating that high KIFC1 expression is associated with increased immune cell infiltration. (D) Scatter plot showing a positive correlation between *KIFC1* expression and clonal neoantigen load in the JAVELIN101 cohort (*R* ^2^ = 0.0329, *p* = 0.0002, *n* = 427), indicating that high KIFC1 expression is associated with increased immune cell infiltration. (E, F) Box plots of CD8^+^ T cell (C) and NK cell (D) infiltration in IMmotion151, stratified by *KIFC1* expression. Tumors with high *KIFC1* expression show significantly higher infiltration of both cell types (**p < 0.01, ****p < 0.0001). (G, H) Box plots of CD8^+^ T cell (E) and NK cell (F) infiltration in JAVELIN101, stratified by KIFC1 expression. High *KIFC1* expression is associated with increased CD8^+^ T cell infiltration (**p < 0.01), while NK cell infiltration shows no significant difference (ns). (I, J) Kaplan–Meier curves for progression‐free survival (PFS) in the IMmotion151 (G) and JAVELIN101 (H) cohorts, comparing outcomes between TKI monotherapy and IO–TKI combination therapy in patients with low (left) and high (right) *KIFC1* expression. In the high *KIFC1* group, combination therapy significantly improves PFS compared to TKI monotherapy, whereas no significant difference is observed in the low *KIFC1* group. These findings suggest that high *KIFC1* expression is associated with an immunologically active tumor microenvironment and may predict improved response to immune checkpoint inhibitor–TKI combination therapy in RCC.

Given the GSEA findings in Figure 3, which highlight the activation of the interferon gamma response in tumors with high *KIFC1* expression, we next examined the relationship between the expression of *KIFC1* and the immune response, as well as the potential therapeutic relevance of ICIs [34, 35, 36]. First, we assessed the correlation between the expression of *KIFC1* and the immune score from the CPTAC cohort. A positive correlation was observed, indicating that high *KIFC1* expression is associated with a more immunologically active tumor microenvironment (*R* ^2^ = 0.0565, *p* = 0.001) (Figure 5A). Similarly, positive correlations were also observed for IMmotion151 (*R* ^2^ = 0.063, *p* < 0.001) and JAVELIN101 (*R* ^2^ = 0.085, *p* < 0.001) (Figure 5B,C). Furthermore, a positive correlation was observed between *KIFC1* expression levels and clonal neoantigen load (*R* ^2^ = 0.0329, *p* = 0.0002), suggesting that high *KIFC1* expression may be associated with an immune‐activated state (Figure 5D). Next, we analyzed immune cell infiltration in relation to the expression of *KIFC1*, focusing on CD8^+^ T cells and NK cells. The analysis revealed a trend toward increased infiltration of both cell types in the high *KIFC1* expression group (Figure 5E–H), further supporting the presence of an active immune microenvironment. In *KIFC1* low‐expression groups, the prognosis remained unchanged regardless of treatment strategy. However, in the high‐expression group, combination therapy with TKIs and ICIs significantly improved patient outcomes (Figure 5I,J). Consistently, across multiple ccRCC cohorts, high *KIFC1* expression was positively correlated with tumor mutational burden (Figure S2A–C). We also evaluated the association between *KIFC1* and immune checkpoint–related signatures: in both the IMmotion151 and JAVELIN101 cohorts, *KIFC1* expression showed significant positive correlations with *PDCD1*(PD‐1) expression (*R* ^2^ = 0.102 and 0.095, respectively), *CD274*(PD‐L1) expression (*R* ^2^ = 0.153 and 0.098), *CTLA4* expression (*R* ^2^ = 0.059 and 0.065), and Treg cell signatures (*R* ^2^ = 0.072 and 0.105; all *p* < 0.001) (Figure S3A–H), indicating that *KIFC1*‐high tumors are characterized by strong activation of immune checkpoint pathways and an immunosuppressive environment. Collectively, these findings suggest that although high *KIFC1* expression is associated with poor prognosis, the incorporation of ICIs may provide therapeutic benefit to this biologically distinct subgroup of ccRCC patients.

### 3.6 Association of *KIFC1* Expression With Renal Cancer‐Associated Genetic Mutations

**FIGURE 6:**
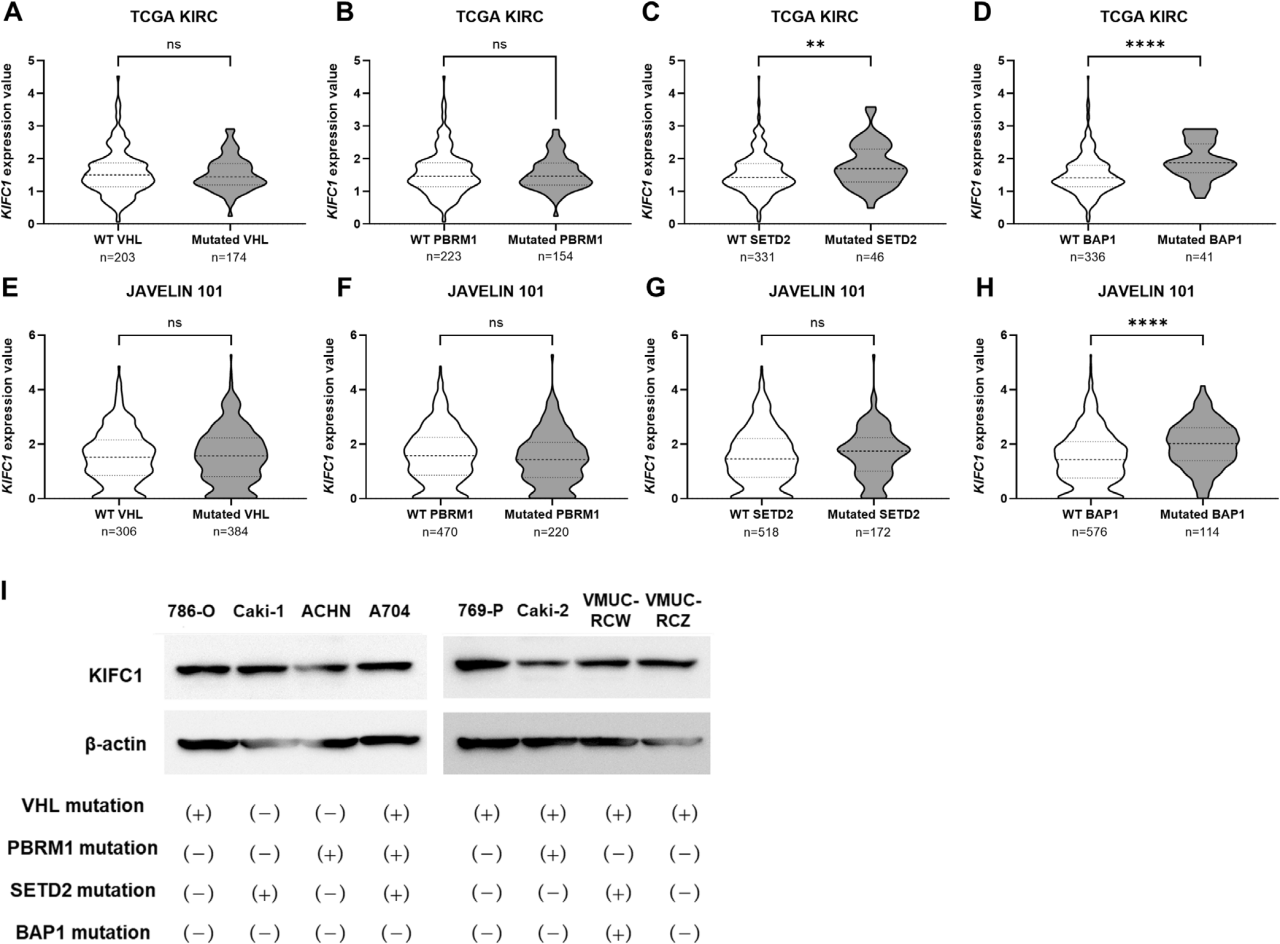
Association of KIFC1 expression with renal cancer‐associated genetic mutations. (A–D) Violin plots showing *KIFC1* expression in TCGA KIRC tumors stratified by mutation status for VHL (A), PBRM1 (B), SETD2 (C), and BAP1 (D). *KIFC1* expression is significantly higher in tumors with SETD2 (**p < 0.01) and BAP1 (****p < 0.0001) mutations, but not with VHL or PBRM1 mutations (ns, not significant). (E–H) Violin plots of *KIFC1* expression in the JAVELIN101 cohort, stratified by mutation status for VHL (E), PBRM1 (F), SETD2 (G), and BAP1 (H). Elevated *KIFC1* expression is observed in tumors with BAP1 mutations (****p < 0.0001), while no significant association is seen with VHL, PBRM1, or SETD2 mutations. (I) We evaluated KIFC1 protein expression in eight renal cell carcinoma cell lines annotated with VHL, PBRM1, SETD2, and BAP1 mutations by Western blot analysis. No significant increase in *KIFC1* expression was observed in VMUC‐RCW cells with BAP1 mutations.

To investigate the relationship between *KIFC1* expression and genetic alterations commonly observed in RCC [37], we analyzed the TCGA KIRC and JAVELIN101 datasets, focusing on mutations in *VHL, PBRM1, SETD2*, and *BAP1*. In the TCGA KIRC cohort, high *KIFC1* expression was significantly associated with mutations in SETD2 and BAP1, while in the JAVELIN101 dataset, high *KIFC1* expression was correlated with BAP1 mutations (Figure 6A–H). In RCC, *BAP1* mutations have been shown to be strongly associated with a high‐grade histology (sarcomatoid differentiation) [38, 39], which is consistent with our analysis (Figure 4). To validate these associations at the protein level, we assessed the *KIFC1* expression in eight RCC cell lines from our institution using western blotting and referenced mutation data from the UCSC Xena database. Notably, increased *KIFC1* expression was observed in VMUC‐RCW cells harboring *BAP1* mutations, consistent with our *in silico* findings. However, this pattern was not consistently observed across all cell lines (Figure 6I).

## 4 Discussion

In this study, we focused on KIFC1, a key molecule involved in centrosome clustering, and comprehensively investigated its expression characteristics and clinical significance using multiple large‐scale cohorts, including IMmotion151, JAVELIN101, and our independent Hiroshima cohort in ccRCC. High *KIFC1* expression was consistently associated with significantly shorter overall survival and progression‐free survival, and multivariate Cox regression analyses demonstrated that it was independently associated with a poor prognosis in the Hiroshima and TCGA KIRC cohorts. Importantly, the expression of *KIFC1* in surgical specimens can be quantitatively assessed by immunohistochemistry. In clinical trials such as KEYNOTE‐564, pathological features including T stage, nuclear grade, and the presence of sarcomatoid features have been used to predict the risk of postoperative recurrence and guide the indication of adjuvant therapy [40]. The expression of *KIFC1* has demonstrated potential utility as a practical biomarker that complements these conventional approaches. Incorporating *KIFC1* expression into risk models may enhance patient stratification and promote the advancement of personalized medicine in ccRCC.

A significant strength of this study is the GSEA performed across multiple independent large‐scale cohorts, as such multi‐cohort analyses are relatively uncommon in ccRCC research. The consistency of analytical results, particularly the repeated enrichment of immune‐related pathways, including the interferon gamma response, and EMT‐related gene sets, underscores the robustness of the biological associations identified. These findings suggest that high *KIFC1* expression is linked to immune and inflammatory pathway activation (especially the interferon gamma response), as well as EMT (a hallmark of sarcomatoid transformation), supporting the notion that *KIFC1* may be a central mediator of both tumor progression and the immune microenvironment.

Consistent with the association with epithelial–mesenchymal transition in GSEA, this study demonstrated that *KIFC1* expression is elevated in sarcomatoid differentiation. These findings are in line with previous reports demonstrating that high *KIFC1* expression is strongly associated with activation of the EMT pathway [41, 42]. Centrosome amplification is known to induce EMT transcription factors such as Snail and ZEB1 via E2F1, leading to cytoskeletal reorganization and loss of cell adhesion through activation of the Myc/Ras pathway [43, 44]. Furthermore, the EMT‐associated enhancement of the RhoA–ROCK pathway and actomyosin contractile force shortens the intercentromeric distance and promotes KIFC1‐dependent centrosome clustering, suggesting a bidirectional positive feedback loop between EMT and centrosome clustering [45]. Recently, attention has also focused on the nuclear function of KIFC1. It has been reported that KIFC1 physically interacts with the chromatin structural factor HMGA1, enhancing its transcriptional activity to drive a group of EMT‐related genes such as STAT3 and MMP2 [32]. Furthermore, TCF‐4, a key transcription factor in the Wnt/β‐catenin pathway, directly binds to the KIFC1 promoter and activates its transcription. This suggests that TCF‐4‐dependent *KIFC1* expression increases following Wnt signaling activation may contribute to the activation of EMT‐related transcriptional programs via HMGA1 and Twist1. Furthermore, KIFC1 has been reported to activate the TGF‐β/SMAD pathway, inducing an EMT phenotype characterized by decreased E‐cadherin and increased N‐cadherin expression [46]. Conversely, *KIFC1* knockdown reduces Snail and ZEB1 protein levels and restores E‐cadherin expression [47], suggesting KIFC1 plays a central role in regulating EMT transcription factors. Furthermore, KIFC1 localizes to the nucleus and cytoplasm during interphase and has been shown to physically support cell migration and invasion by interacting with non‐muscle myosin IIA (MYH9) [33]. These findings suggest that KIFC1 is a multifunctional molecule that comprehensively supports the morphological changes and acquisition of motility associated with EMT, acting not only as a microtubule regulator but also as a cytoskeletal control factor involving the actomyosin contractile system. From a therapeutic resistance perspective, it has been reported that *KIFC1* overexpression activates the PI3K/AKT pathway and contributes to TKI resistance [48]. The inverse correlation between *KIFC1* expression and angiogenesis activity observed in this study suggests that tumors with high *KIFC1* expression may be poor targets for angiogenesis inhibitors, potentially limiting the efficacy of TKI therapy.

In terms of the immune microenvironment, tumors with high *KIFC1* expression exhibited immunologically active features, including high immune scores, increased infiltration of CD8^+^ T cells, and a high clonal neoantigen load. High immune scores, increased CD8^+^ T‐cell infiltration, and sarcomatoid features are generally considered favorable indicators for ICI responsiveness [49, 50, 51], and significant clinical benefit from ICIs has been reported in RCC with sarcomatoid features [52]. Consistent with these observations, we found that patients with high *KIFC1* expression derived greater clinical benefit from ICI‐containing combination therapy than from TKI monotherapy, suggesting that *KIFC1*‐associated treatment resistance can be at least partially overcome by incorporating immunotherapy. Moreover, previous reports indicate that *KIFC1* expression is correlated with microsatellite instability and tumor mutational burden in other malignancies [53], implying a molecular background that favors immunotherapy responsiveness, a notion supported by our findings in ccRCC. Tumors with a high clonal neoantigen load are more likely to elicit a strong CD8^+^ T‐cell response, and reactivation via *PDCD1* or *CTLA4* blockade has been shown to be effective, supporting its utility as a prognostic biomarker for ICIs [54]; the positive correlation observed between *KIFC1* expression and clonal neoantigen load further reinforces the potential immunogenicity of *KIFC1*‐high tumors. KIFC1 has been reported to induce chromosomal instability (CIN) through chromosome segregation errors and micronucleus formation [55]. Furthermore, it is known that when micronucleus‐derived DNA leaks into the cytoplasm due to CIN, the cGAS–STING pathway is activated, inducing immune cell infiltration via the production of inflammatory cytokines and type I interferons [56, 57]. Based on these findings, the immunologically active state observed in this study may be explained by KIFC1‐dependent CIN progression followed by activation of the innate immune response via the cGAS–STING pathway. At the same time, however, several reports suggest that the efficacy of ICI monotherapy is limited despite high *KIFC1* expression [58, 59]. Our data provide a potential explanation for this paradox: *KIFC1* expression positively correlated not only with *PDCD1* and *CD274*, but also with *CTLA4* and regulatory T‐cell infiltration, indicating the coexistence of strong immune activation and potent immunosuppressive signaling within the *KIFC1*‐high TME. Together with previous reports showing that *KIFC1*‐high tumors upregulate T‐cell exhaustion and bystander markers (e.g., CD38, CD101, TIM‐3, LAG‐3, TOX, and TIGIT) [53], these findings suggest that *KIFC1* may contribute to a “qualitative immunodeficiency” state in which abundant but functionally impaired T cells fail to mount an effective antitumor response, thereby limiting the efficacy of ICI monotherapy. In this context, targeting *KIFC1* to restore antigen presentation and relieve immune suppression, or combining ICIs that block both *PDCD1/CD274* and *CTLA4* pathways, may further enhance the therapeutic benefit in *KIFC1*‐high ccRCC and represents an attractive strategy for future combination immunotherapy.

In recent years, the importance of interactions not only with tumor cells but also with the tumor microenvironment (TME) has been emphasized. The cellular composition and immunological heterogeneity within the TME of ccRCC have been reported to critically determine tumor progression and responsiveness to systemic therapies, including ICIs [60, 61]. In particular, features of the multilayered immune microenvironment, such as immunosuppressive cell populations, cytokine/chemokine signatures, and metabolic reprogramming, have garnered attention as predictive biomarkers for ICI response [62, 63, 64]. In this study's GSEA, high *KIFC1* expression was consistently associated with activation of immune/inflammatory pathways centered on the interferon gamma response and with EMT, a hallmark of sarcomatoid transformation. However, this analysis is based on bulk data combining tumor cells and stroma, and does not clearly demonstrate a direct relationship between KIFC1 and TME. On the other hand, it suggests that KIFC1 functions as a biomarker reflecting both tumor cell‐specific malignant properties (EMT and proliferation) and immunological features of the TME, potentially acting as a central mediator linking tumor progression and the immune microenvironment. The role of KIFC1 in the ccRCC TME remains incompletely understood. Future research should employ single‐cell RNA sequencing and spatial transcriptomics to elucidate the origin of *KIFC1*‐expressing cells and their interactions with TME constituent cells. This will pave the way for studies aiming to apply these insights to personalized treatment strategies based on TME heterogeneity.

This study has several limitations that should be acknowledged. First, *KIFC1* expression was evaluated by immunohistochemistry on surgical resection specimens, and cases with ≥ 10% of tumor cells showing positive staining were defined as having high *KIFC1* expression. This cutoff was selected in an exploratory manner based on the distribution within our cohort and prior reports, and it has not yet been validated for reproducibility or interinstitutional robustness as a clinical grading criterion. Second, this study is primarily based on retrospective and in silico analyses and does not experimentally demonstrate that *KIFC1* overexpression causally induces EMT, immune activation, or resistance to TKIs. Although previous studies have shown that *KIFC1* knockdown in RCC and other cancer cell lines reduces proliferation, colony formation, invasion, and migration and induces G2/M cell cycle arrest, functional validation using ccRCC cell lines and in vivo models is still required to clarify the direct impact of *KIFC1* on tumor biology and to elucidate upstream regulatory mechanisms at the transcriptional, epigenetic, and noncoding RNA levels. Third, although we leveraged multiple large‐scale public datasets, the cohorts used in this study had limited publicly available clinical information, and the depth of clinical annotation varied. Consequently, we could not fully adjust for established prognostic factors in multivariable analyses, and technical variability may limit the generalizability of inter‐cohort comparisons and conclusions regarding treatment response. Therefore, to establish *KIFC1* as an independent predictor of immunotherapy response, validation in a well‐annotated prospective cohort with more detailed clinical information is required. Finally, our *KIFC1* assessment is tissue‐based and depends on surgically resected specimens. In contrast, recent advances in liquid biopsy have highlighted circulating biomarkers, including blood microRNAs, as promising tools for noninvasive diagnosis, prognostication, and treatment response monitoring in ccRCC [65]. Identifying circulating biomarkers that correlate with *KIFC1* expression and capture *KIFC1*‐related molecular subtypes could substantially enhance the clinical applicability of our findings and facilitate individualized treatment planning in the future.

## 5 Conclusion

This study demonstrates that high *KIFC1* expression in ccRCC is associated with a poor prognosis, EMT, therapeutic resistance, and the development of an immunosuppressive tumor microenvironment. These findings highlight *KIFC1* as a multifunctional molecule that drives tumor progression and facilitates immune evasion. As a potential immunohistochemical biomarker, *KIFC1* may assist in prognostic assessment and in predicting responses to TKIs and ICIs. Furthermore, *KIFC1* may represent a promising therapeutic target. Future research should aim to elucidate the underlying mechanisms and evaluate the efficacy of *KIFC1*‐targeted therapies, particularly in combination with ICIs, to improve clinical outcomes in ccRCC.

## Author Contributions

Yohei Sekino contributed to conceptualization. Methodology was performed by Yohei Sekino, Go Kobayashi, Hikaru Nakahara, Shintaro Akabane, and Akihiro Goriki. Investigation was carried out by Yoshinori Nakano, Go Kobayashi, Hikaru Nakahara, Hiroyuki Kitano, and Keisuke Goto. Validation was performed by Yoshinori Nakano, Kenshiro Takemoto, Miki Naito, Hiroyuki Kitano, Keisuke Goto, Akihiro Goriki, and Keisuke Hieda. Visualization was contributed by Shunsuke Miyamoto, Kohei Kobatake, and Keisuke Hieda. Project administration was conducted by Takao Hinoi and Nobuyuki Hinata. Supervision was provided by Nobuyuki Hinata. Funding acquisition was secured by Yoshinori Nakano and Yohei Sekino. Yoshinori Nakano drafted the original manuscript. Yohei Sekino reviewed and edited the manuscript. All authors read and approved the final manuscript.

## Funding

This work was supported by the Japan Society for the Promotion of Science (19K18586).

## Ethics Statement

This study was approved by the Institutional Review Board of Hiroshima University (Approval Number: [E‐588‐2]). All procedures were conducted in accordance with the Declaration of Helsinki.

## Consent

Informed consent was waived due to the retrospective nature of the study and the use of anonymized data, in accordance with the regulations of the Institutional Review Board of Hiroshima University.

## Conflicts of Interest

The authors declare no conflicts of interest.

## Supporting information


**Appendix S1:** Supplementary methods.

**Figure S1:** Correlation of KIFC1 expression with EMT‐related genes in TCGA KIRC. (A) Scatter plot showing the correlation between KIFC1 and HMGA1 expression in the TCGA KIRC cohort (slope = 0.29, *R* ^2^ = 0.089, *p* < 0.001; *n* = 510). (B) Scatter plot showing the correlation between KIFC1 and MYH9 expression in the TCGA KIRC cohort (slope = 0.23, *R* ^2^ = 0.058, *p* < 0.001; *n* = 510).

**Figure S2:** Association between KIFC1 expression and tumor mutational burden (TMB) across ccRCC cohorts. Scatter plots showing the relationship between KIFC1 expression and TMB in (A) TCGA KIRC (slope = 0.0649, *R* ^2^ = 0.013, *p* = 0.022; *n* = 400), (B) IMmotion151 (slope = 0.063, *R* ^2^ = 0.023, *p* < 0.001; *n* = 670), and (C) JAVELIN101 (slope = 0.0062, *R* ^2^ = 0.038, *p* < 0.0001; *n* = 583).

**Figure S3:** Correlations between KIFC1 expression and immune checkpoint–related markers and Treg signatures in ICI‐treated ccRCC cohorts. Scatter plots showing correlations between KIFC1 expression and immune checkpoint–related gene expression/signatures in IMmotion151: (A) PDCD1 (PD‐1) (slope = 0.00095, *R* ^2^ = 0.102, *p* < 0.001; *n* = 823), (B) CD274 (PD‐L1) (slope = 0.0011, *R* ^2^ = 0.153, *p* < 0.001; *n* = 823), (C) CTLA4 (slope = 0.0015, *R* ^2^ = 0.059, *p* < 0.001; *n* = 823), and (D) Treg cell signatures (slope = 0.0018, *R* ^2^ = 0.072, *p* < 0.001; *n* = 823); and in JAVELIN101: (E) PDCD1 (PD‐1) (slope = 0.0009, *R* ^2^ = 0.095, *p* < 0.001; *n* = 726), (F) CD274 (PD‐L1) (slope = 0.0013, *R* ^2^ = 0.098, *p* < 0.001; *n* = 726), (G) CTLA4 (slope = 0.0013, *R* ^2^ = 0.065, *p* < 0.001; *n* = 726), and (H) Treg cell signatures (slope = 0.0010, *R* ^2^ = 0.105, *p* < 0.001; *n* = 726).

**Table S1:** Data sources.

## Data Availability

The data that supports the findings of this study are available in the Supporting Information of this article.
