# Supplementary material for: KIFC1 is Associated With Sarcomatoid Differentiation, Immune Response, and a Poor Prognosis in Clear Cell Renal Cell Carcinoma
Source: Cancer Med. 2026 Feb 26;15(3):e71687. doi: 10.1002/cam4.71687 (PMC12945555; doi:10.1002/cam4.71687)
Supplement: Supplementary file 1 — Appendix S1: Supplementary methods. [file CAM4-15-e71687-s003.docx]

**Supplementary Methods**

**Immunohistochemistry**

IHC was performed on one or two representative tumor blocks using a Bond-3 automated immunostainer platform (Leica Biosystems, Bannockburn, IL, USA) according to the manufacturer’s protocol. Antigen retrieval was performed using heat-based antigen retrieval citrate-based low pH epitope retrieval buffer (pH 6; Leica ER1 buffer) with a heating time of 20 min at 99°C. Peroxidase activity was blocked for 5 min with a Bond Polymer Reagent Kit. The sections were incubated with a mouse polyclonal anti-KIFC1 antibody (1:100, H00003833-M01, Abnova, Taipei, Taiwan) for 30 min. Stain detection was performed using a Bond Polymer Detection Kit (Bond-3, Leica Biosystems) for 8 min. The sections were incubated with DAB for 10 min for color reactions and then counterstained with 0.1% hematoxylin from a Bond Polymer Detection Kit. When >10% of the tumor cells were stained, the sample was considered positive for KIFC1 (according to the median cutoff values rounded to the nearest 10%).

**Statistical Analysis**

All experiments were repeated at least three times, with each sample tested in triplicate. The results are expressed as the mean ± standard deviation of triplicate measurements. The sample sizes for the relevant experiments were determined by a power analysis. Statistical differences were evaluated using the two-tailed Student’s t test or Mann‒Whitney U test. One-way analysis of variance (ANOVA) was used to determine statistically significant differences. P values of <0.05 were considered to indicate statistical significance. After a Kaplan‒Meier analysis, the significance of differences between the survival curves of the cohorts was determined with a log-rank Mantel‒Cox test. Statistical analyses were primarily conducted using GraphPad Prism 10 (GraphPad Software Inc., San Diego, CA, USA) or JMP Pro 17 (SAS Institute, Cary, NC, USA).
