# Supplementary figures and images for: KIFC1 is Associated With Sarcomatoid Differentiation, Immune Response, and a Poor Prognosis in Clear Cell Renal Cell Carcinoma
Source: Cancer Med. 2026 Feb 26;15(3):e71687. doi: 10.1002/cam4.71687 (PMC12945555; doi:10.1002/cam4.71687)

Supplementary Figure.S1

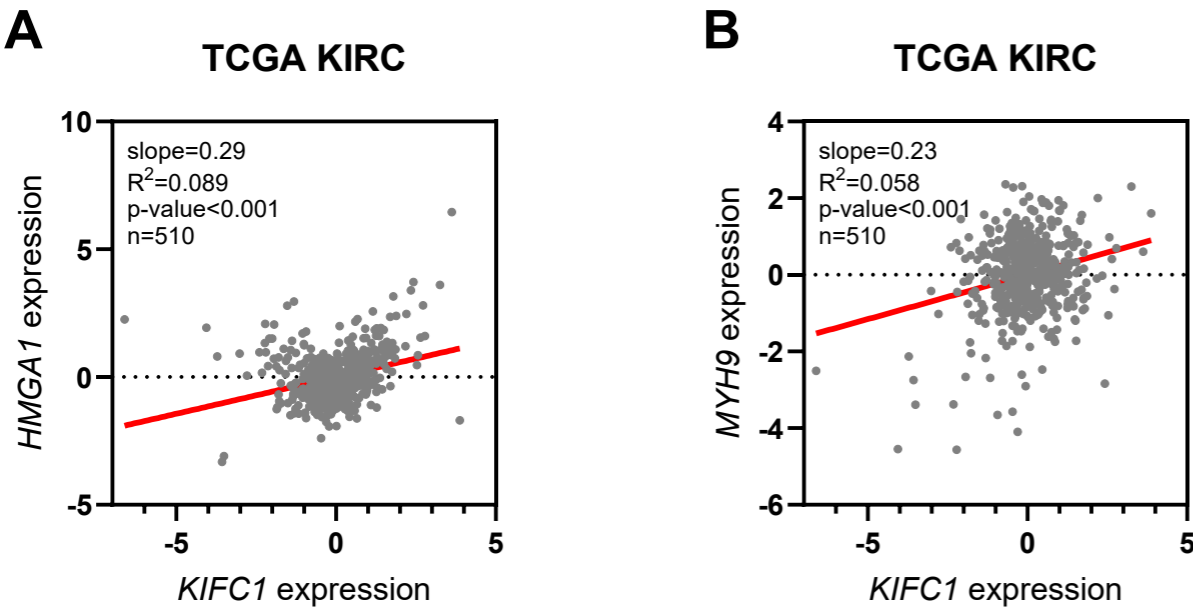

Supplement: Supplementary file 2 — Figure S1: Correlation of KIFC1 expression with EMT‐related genes in TCGA KIRC. (A) Scatter plot showing the correlation between KIFC1 and HMGA1 expression in the TCGA KIRC cohort (slope = 0.29, R 2 = 0.089, p < 0.001; n = 510). (B) Scatter plot showing the correlation between KIFC1 and MYH9 expression in the TCGA KIRC cohort (slope = 0.23, R 2 = 0.058, p < 0.001; n = 510). [file CAM4-15-e71687-s004.pdf]

Supplementary Figure.S2

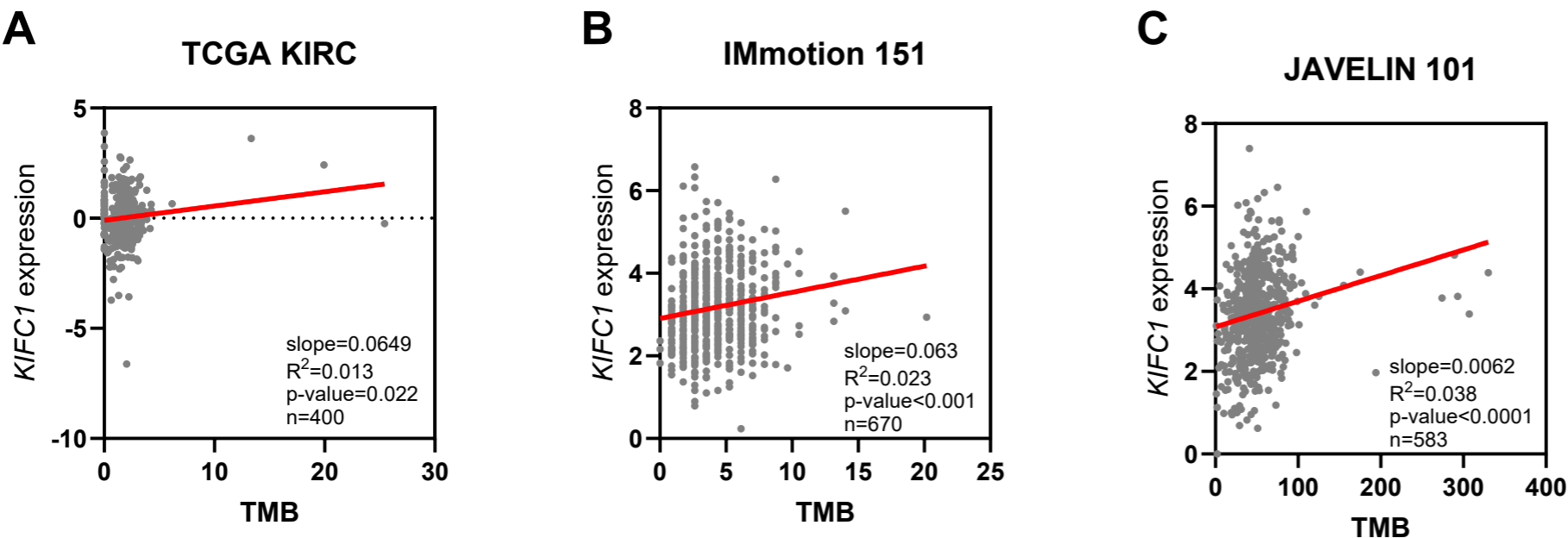

Supplement: Supplementary file 3 — Figure S2: Association between KIFC1 expression and tumor mutational burden (TMB) across ccRCC cohorts. Scatter plots showing the relationship between KIFC1 expression and TMB in (A) TCGA KIRC (slope = 0.0649, R 2 = 0.013, p = 0.022; n = 400), (B) IMmotion151 (slope = 0.063, R 2 = 0.023, p < 0.001; n = 670), and (C) JAVELIN101 (slope = 0.0062, R 2 = 0.038, p < 0.0001; n = 583). [file CAM4-15-e71687-s001.pdf]

Supplementary Figure.S3

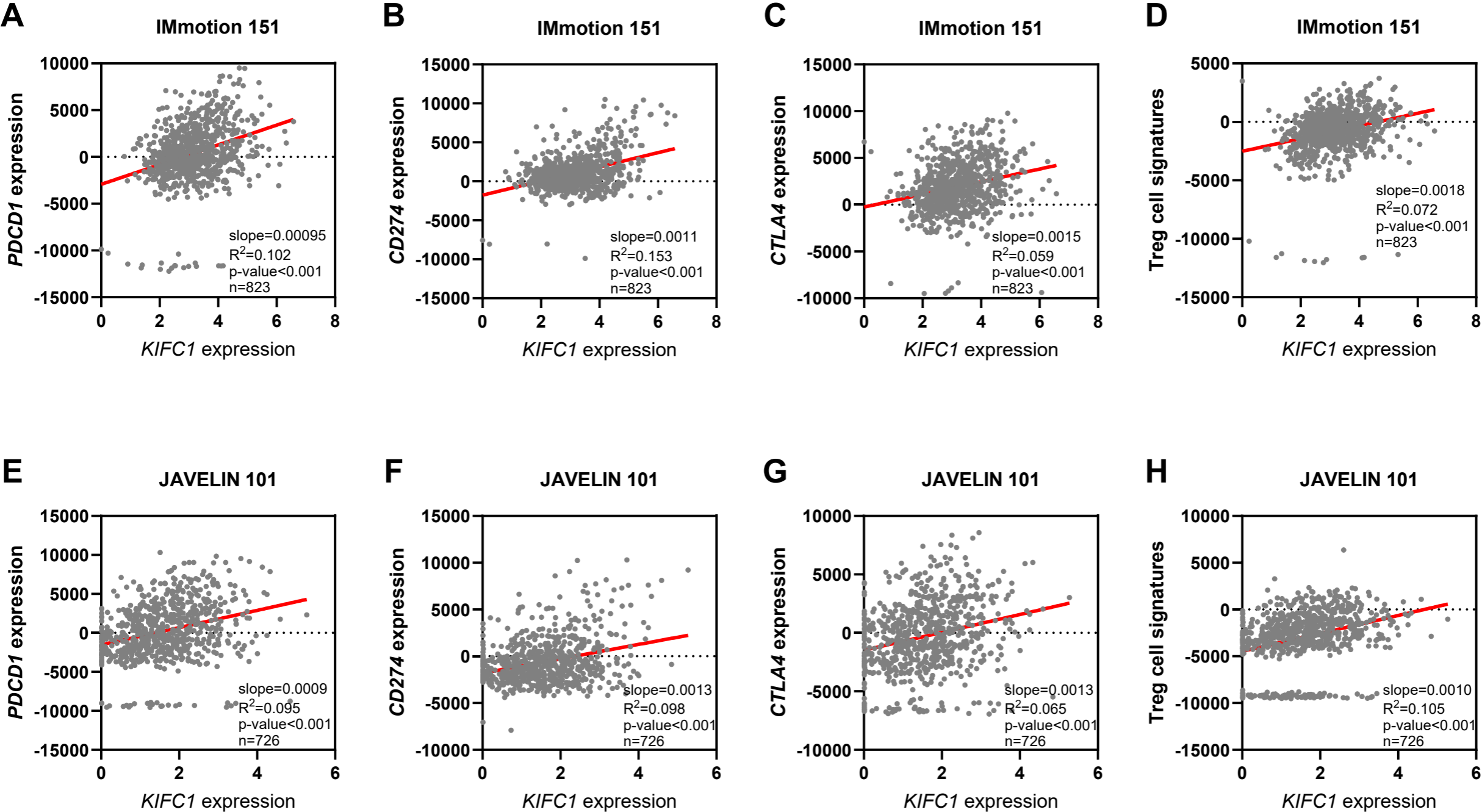

Supplement: Supplementary file 4 — Figure S3: Correlations between KIFC1 expression and immune checkpoint–related markers and Treg signatures in ICI‐treated ccRCC cohorts. Scatter plots showing correlations between KIFC1 expression and immune checkpoint–related gene expression/signatures in IMmotion151: (A) PDCD1 (PD‐1) (slope = 0.00095, R 2 = 0.102, p < 0.001; n = 823), (B) CD274 (PD‐L1) (slope = 0.0011, R 2 = 0.153, p < 0.001; n = 823), (C) CTLA4 (slope = 0.0015, R 2 = 0.059, p < 0.001; n = 823), and (D) Treg cell signatures (slope = 0.0018, R 2 = 0.072, p < 0.001; n = 823); and in JAVELIN101: (E) PDCD1 (PD‐1) (slope = 0.0009, R 2 = 0.095, p < 0.001; n = 726), (F) CD274 (PD‐L1) (slope = 0.0013, R 2 = 0.098, p < 0.001; n = 726), (G) CTLA4 (slope = 0.0013, R 2 = 0.065, p < 0.001; n = 726), and (H) Treg cell signatures (slope = 0.0010, R 2 = 0.105, p < 0.001; n = 726). [file CAM4-15-e71687-s005.pdf]
